# Stabilization of IGF2BP1 by USP10 promotes breast cancer metastasis via CPT1A in an m6A-dependent manner

**DOI:** 10.7150/ijbs.76798

**Published:** 2023-01-01

**Authors:** Jiajun Shi, Qianyi Zhang, Xi Yin, Jiahui Ye, Shengqing Gao, Chen Chen, Yaxuan Yang, Baojuan Wu, Yuping Fu, Hongmei Zhang, Zhangding Wang, Bo Wang, Yun Zhu, Hongyan Wu, Yongzhong Yao, Guifang Xu, Qiang Wang, Shouyu Wang, Weijie Zhang

**Affiliations:** 1 Department of General Surgery, Nanjing Drum Tower Hospital, The Affiliated Hospital of Nanjing University Medical School, Nanjing 210000, Jiangsu Province, People's Republic of China.; 2 Jiangsu Key Laboratory of Molecular Medicine, Medical School of Nanjing University, Nanjing 210000, Jiangsu Province, People's Republic of China.; 3 Department of Neurosurgery, Jinling Hospital, Medical School of Nanjing University, Nanjing 210000, Jiangsu Province, People's Republic of China.; 4 Nanjing Drum Tower Hospital Clinical College of Traditional Chinese and Western Medicine, Nanjing University of Chinese Medicine, Nanjing 210000, Jiangsu Province, People's Republic of China.; 5 Department of Pathology, Nanjing Drum Tower Hospital, The Affiliated Hospital of Nanjing University Medical School, Nanjing 210000, Jiangsu Province, People's Republic of China.; 6 Department of Gastroenterology, Nanjing Drum Tower Hospital, The Affiliated Hospital of Nanjing University Medical School, Nanjing 210000, Jiangsu Province, People's Republic of China.; 7 Department of Hepatobiliary Surgery, Nanjing Drum Tower Hospital, The Affiliated Hospital of Nanjing University Medical School, Nanjing 210000, Jiangsu Province, People's Republic of China.; 8 Department of Hepatobiliary Surgery, The First Affiliated Hospital of Anhui Medical University, Hefei 230022, Anhui Province, People's Republic of China.; 9 Center for Public Health Research, Medical School of Nanjing University, Nanjing 210000, Jiangsu Province, People's Republic of China.

**Keywords:** Breast cancer, metastasis, m6A, IGF2BP1, USP10, CPT1A

## Abstract

Metastasis leads to the vast majority of breast cancer mortality. Increasing evidence has shown that N6-methyladenosine (m6A) modification and its associated regulators play a pivotal role in breast cancer metastasis. Here, we showed that overexpression of the m6A reader IGF2BP1 was clinically correlated with metastasis in breast cancer patients. Moreover, IGF2BP1 promoted distant metastasis *in vitro* and *in vivo*. Mechanistically, we first identified USP10 as the IGF2BP1 deubiquitinase. USP10 can bind to, deubiquitinate, and stabilize IGF2BP1, resulting in its higher expression level in breast cancer. Furthermore, by MeRIP-seq and experimental verification, we found that IGF2BP1 directly recognized and bound to the m6A sites on CPT1A mRNA and enhanced its stability, which ultimately mediated IGF2BP1-induced breast cancer metastasis. In clinical samples, USP10 levels correlated with IGF2BP1 and CPT1A levels, and breast cancer patients with high levels of USP10, IGF2BP1, and CPT1A had the worst outcome. Therefore, these findings suggest that the USP10/IGF2BP1/CPT1A axis facilitates breast cancer metastasis, and this axis may be a promising prognostic biomarker and therapeutic target for breast cancer.

## Introduction

Breast cancer (BC) has become the most commonly diagnosed cancer (11.7%) among all cancer cases, surpassing lung cancer (11.4%) for females in 2020 1. Moreover, female BC was the fifth leading cause of cancer death worldwide (6.9%) 1. Metastasis occurs in more than 90% of all BC-related deaths 2-5. The lungs, bones, brain, and liver are the most favourable BC metastatic sites 6-8. However, the molecular mechanism of BC metastasis remains unknown.

Epigenetic reprogramming is an important feature of BC cells with metastatic competence 9. N6-Methyladenosine (m6A) modification is universally accepted to be the most pervasive and abundant RNA modification in eukaryotic RNAs 10 and has drawn much attention in recent years 11. Increasing evidence has shown that m6A modification plays a pivotal role in cancer metastasis by regulating RNA metabolism in tumour cells, cancer stem cells 12, tumour-associated macrophages 13, and others 10, 14. The function of m6A modification has been widely reported in different cancers, including BC 15, gastrointestinal cancer (GC) 16, 17, ovarian cancer (OC) 18, and hepatocellular carcinoma (HCC) 12. The methyltransferase complexes (“writers”) and demethylases (“erasers”) are responsible for the dynamic changes in m6A in specific transcripts. Methyltransferase complexes, including methyltransferase-like 3 (METTL3), METTL14, Wilms tumour 1-associated protein (WTAP), RBM15/15B, VIRMA (also called KIAA1429), and ZC3H13, catalyse the formation of m6A 19. Demethylases, including fat mass and obesity-associated protein (FTO) and alkB homologue 5 (ALKBH5), selectively remove m6A from RNAs. Moreover, RNA-binding proteins (“readers”), including YTH domain-containing proteins (YTHDC1/2, YTHDF1/2/3), eukaryotic initiation factor 3 (eIF3), the IGF2 mRNA binding protein (IGF2BP1/2/3) families and the heterogeneous nuclear ribonucleoprotein (HNRNP) protein families, could affect the translation, splicing, exportation and stability of RNA by binding to and recognizing the m6A sites on RNA 20-22. In particular, the m6A reader IGF2BP1 has been reported to function by promoting the stability of RNAs and upregulating numerous oncogenes dependent on m6A methylation 23-26. Recently, the role of IGF2BP1 in the pathogenesis of BC metastasis has attracted much attention 27.

Ubiquitination, as a posttranslational modification, not only takes part in a large number of physiological events but also facilitates the origin and progression of cancer 28. To date, the number of identified deubiquitinases (DUBs) is more than 100 29. By removing ubiquitin (Ub) from the substrate, DUBs can rescue the specific protein from being marked for degradation and maintain its protein level 28. The deregulation of some DUBs is involved in the metastasis of BC 30-32. Recently, the degradation of IGF2BPs has been reported to be related to the ubiquitin‒proteasome system. However, the DUBs of IGF2BPs have not yet been discovered 33, 34.

In our study, we report the oncogenic role of IGF2BP1 in BC metastasis and identified USP10 as the deubiquitinase of IGF2BP1 for the first time. Moreover, we identified CPT1A, which has been reported to endow BC cells with the potential for metastasis 35-37, as the target gene of IGF2BP1. Based on our clinical prognostic data, BC patients with high expression levels of USP10, IGF2BP1, and CPT1A had the worst outcome. Therefore, our study uncovers an essential role of the USP10/IGF2BP1/CPT1A axis in the regulation of BC metastasis and provides a promising prognostic biomarker and therapeutic target for BC patients with metastasis.

## Materials and Methods

### Patients and clinical samples

Examination of the signatures on the informed consent forms was exempted following the rules approved by the Ethics Committees of Nanjing Drum Towel Hospital and the Affiliated Hospital of Nanjing University Medical School. The cases in which patients were simultaneously suffering from other malignant diseases had previously been ruled out. In this study, 80 paraffin-embedded tissues obtained from the Department of Pathology of Nanjing Drum Hospital after BC resection from 2010 to 2019 were used. Two pathologists individually confirmed the results of haematoxylin and eosin (HE) staining and immunohistochemical (IHC) staining. The ages of the patients ranged from 27 to 74 years. The latest follow-up date was December 21, 2019.

### Cell lines and cell culture

Human HEK293T, MDA-MB-468 and HCC1806 cells were obtained from the Shanghai Institute of Biochemistry and Cell Biology. The corresponding results with MDA-MB-231 cells and metastatic derivative cells LM2, BM6, and 1833 were described in a previous study 38. HCC1806 cells were cultured in 1640 medium supplemented with 10% foetal bovine serum (FBS) and penicillin‒streptomycin solution (Biochannel, Nanjing, China). Other cell lines were cultured in DMEM supplemented with 10% foetal bovine serum (FBS) and penicillin‒streptomycin solution (Biochannel, Nanjing, China). All cells were cultured in an incubator with 5% CO_2_ at 37 °C. Cells were stored at -80 °C using CELLSAVING (New Cell & Molecular Biotech, Suzhou, China).

### Dot blot assay

This experiment followed the protocol in the bio-protocol database (https://bio-protocol.org/e2095). Briefly, mRNA isolated from the indicated cells was further denatured and then spotted onto a Hybond-N+ membrane and crosslinked by UV Crosslinker. After blocking the membrane, an anti-m6A antibody (1:1000; Abcam, USA) was incubated with the membrane overnight at 4 °C. Then, the membrane was further incubated with an HRP-conjugated anti-mouse antibody. Finally, images were recorded with a CCD camera (Tanon, Shanghai, China). Methylene blue (MB) was used to interact with mRNA and also used for normalization.

### Transfection and lentiviral transduction

siRNAs targeting USP10 and CPT1A were designed and synthesized by Genomeditech (Shanghai, China). Expressing plasmids, including USP1, USP2, USP3, USP4, USP5, USP7, USP8, USP10, USP11, USP12, USP13, USP14, USP15, USP16, USP17, USP18, USP20, USP21, USP22, USP26, USP28, USP32, USP33, USP36, USP37, USP38, USP39, USP43, USP44, USP45, USP46, USP47, USP48, USP49, USP50, USP51, USP52, USP54, COPS5, YOD1, BRCC3, EIF3F, VCPIP1, IGF2BP1 and CPT1A, with the indicated Flag- or HA-tags were constructed by Youbio (Hunan, China). Transfection of siRNA and plasmids was conducted using Lipofectamine 3000 (Invitrogen, USA). All siRNA sequences are listed in Supplementary Table 1.

Lentiviral plasmids carrying shIGF2BP1, shUSP10 and USP10 cDNA were designed and cloned into PGMLV-hU6-MCS-CMV-Luciferase-PGK-Puro, Cas9-Puro-U6 and PGMLV-CMV-MCS-3×Flag-EF1-ZsGreen1-T2A-Blasticidin, respectively, by Genomeditech (Shanghai, China). Forty-eight hours after lentiviral infection, cells were further selected with 2 μg/ml puromycin and 6 μg/ml blasticidin (Gibco). All target sequences are listed in Supplementary Table 1.

### Mouse model of BC lung metastasis

Female BALB/c nude mice (4-5 weeks, 18-20 g) were purchased from GemPharmatech (Nanjing, China) and raised in a specific pathogen-free (SPF) experimental animal room. Treated cells (1×10^6^/100 μl PBS) were injected through the tail vein of the nude mice. Pulmonary metastasis was evaluated by bioluminescence imaging at 4 or 8 weeks. Then, the mice were sacrificed, and the lung tissues were imaged and fixed in 4% paraformaldehyde for further analyses.

### RNA isolation and quantitative real-time PCR (qRT-PCR)

Total RNA was isolated from cultured cells using TRIzol reagent (Invitrogen), and reverse transcription was carried out by HiScript II Q RT SuperMix for qPCR (+gDNA wiper) (Vazyme) following the protocol provided by the manufacturer. The primers involved in the qRT‒PCR experiments are listed in Supplementary Table 2. qRT-PCR was performed with 2 × ChamQ SYBR qPCR Green Master Mix (Vazyme Biotech Co., Ltd., Nanjing, China). The results were calculated by the 2 -ΔΔCt formula and normalized to the GAPDH levels.

### RNA immunoprecipitation (RIP)

A MagnaRIP RNA-Binding Protein Immunoprecipitation Kit (Millipore, MA, USA) was used following the manufacturer's instructions. The cell lysates were incubated with beads coated with 5 µg of control mouse IgG or anti-m6A antibody (Abcam, MA, USA) with gentle rotation at 4 °C overnight. Then, specifically captured products were obtained for the detection of CPT1A expression by qRT-PCR. The primers involved in the qRT-PCR experiments are listed in Supplementary Table 2.

### Haematoxylin and eosin (HE) and immunohistochemical (IHC) staining

HE and IHC staining followed the standard protocols described in a previous study 39. The results of IHC staining were evaluated considering both the staining intensity and proportion of tumour cells. The intensity was evaluated as follows: 0, negative; 1, weak; 2, moderate; and 3 strong. The proportion score was determined as follows: 0, less than 5%; 1, 5% to 25%; 2, 26% to 50%; 3, 51% to 75%; and 4, greater than 75%. The final IHC staining index was calculated by multiplying the two numbers (the final values ranged from 0 to 12).

### Immunofluorescence (IF) assay

The indicated cells were seeded in a 24-well plate at a density of 2 × 10^4^ cells per well. After 24 h, the cells were fixed with methanol for 15 min at room temperature and treated with 0.3% Triton X-100 for 15 min. After blocking for 30 min at room temperature, the indicated antibodies were added to the plate separately for incubation overnight at 4 °C. After being washed with PBS 3 times, the plate was further incubated with the secondary antibodies for 2 h at room temperature in the darkroom. DAPI was used to stain the nuclei. Finally, the cells were observed and imaged by fluorescence microscopy (LEICA DMi8, Germany).

### Western blotting assay

Western blotting assays were performed according to standard protocols. Briefly, RIPA lysis buffer (P0013B, Beyotime) supplemented with protease inhibitor cocktail (P1005, Beyotime) and PMSF (ST507-10 ml, Beyotime) was used to lyse cells for 30 min at 4 °C. After centrifugation, the supernatants were collected. The protein concentration was also determined before western blotting. After resolving the proteins by SDS-polyacrylamide electrophoresis, the proteins were transferred to PVDF membranes. After the blotted membranes were blocked for 1 h, they were incubated with the indicated primary antibody overnight at 4 °C and then incubated with HRP-conjugated secondary antibodies for 1 h. Finally, the results were recorded by a CCD camera (Tanon, Shanghai, China) by ECL chemiluminescence (Millipore, Billerica, MA, USA). All antibodies are listed in Supplementary Table 3.

### Transwell assay

The invasion and migration abilities of the cells were evaluated using Transwell chambers with 8 µm pore filters. After 48 h of transfection, cells (3 × 10^4^/100 µl) were seeded on the upper chambers coated with or without 70 µl of Matrigel (BD Biosciences) in serum-free DMEM. DMEM containing 10% FBS was added to the lower chambers. After incubation for 24 h at 37 °C, the cells in the chambers were collected and fixed with methanol or 4% paraformaldehyde. After removing the nonmigrating or noninvading cells with cotton swabs, the chambers were stained with crystal violet solution for 20 min. The results were recorded at ×200 magnification under a microscope. All assays were repeated three times in duplicate.

### RNA-seq and MeRIP-seq

RNA-seq and MeRIP-seq were performed by Gene Denovo (Guangzhou, China). Briefly, after extracting total RNA from LM2 cells with stable IGF2BP1 knockdown and the corresponding control cells, RNA was immunoprecipitated with an anti-m6A antibody and further used to construct RNA libraries, which were sequenced using Illumina NovaSeq^TM^ 6000 by Gene Denovo Biotechnology Co. (Guang Zhou, China).

### Statistical analysis

A paired t test or Wilcoxon signed-rank test was used to evaluate the mRNA levels of the major m6A-related enzymes in 112 breast cancer samples and the paired normal tissues from TCGA database. The differences in IGF2BP1 IHC staining indices in 11 primary breast tumours and the paired normal breast tissues were assessed by the Wilcoxon signed-rank test (grouped). Kaplan-Meier survival curves were analysed by the log-rank test. The correlations between the IHC staining indices of USP10 and IGF2BP1 were analysed by Spearman's test. Student's t test or conventional one-way ANOVA was used to identify statistically significant differences (P < 0.05) between the treated and control groups. GraphPad PRISM 8.0 was used to analyse all the data (GraphPad Software, La Jolla, CA, USA). All experiments were repeated three times. Data are shown as the mean ± SD.

## Results

### Elevated IGF2BP1 expression resulted in poor prognosis of BC

**Figure 1:**
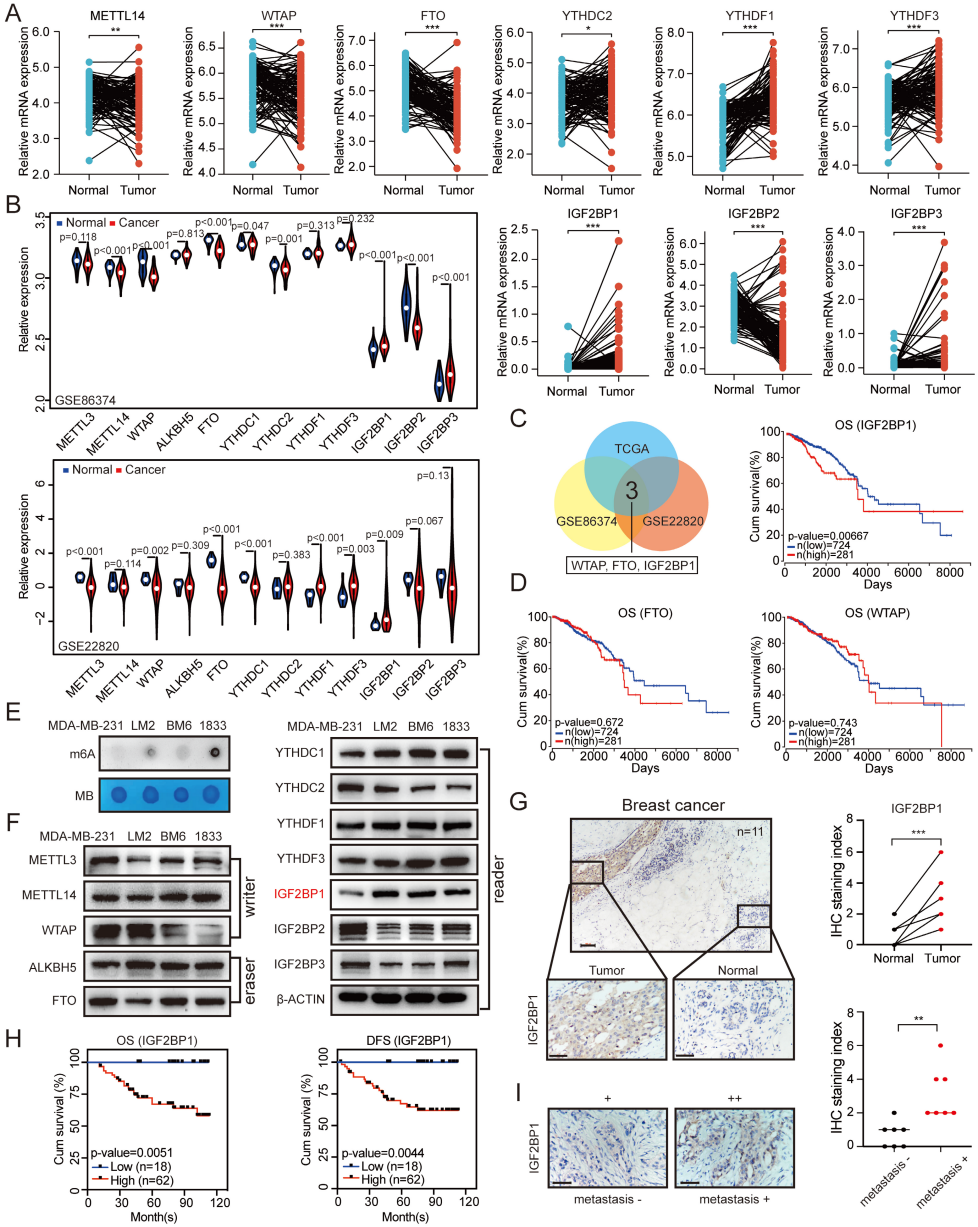
** Elevated IGF2BP1 expression resulted in poor prognosis of BC. (A)** The mRNA levels of m6A-related enzymes in 112 BC tissues and paired normal breast tissues from TCGA database (n=112, p<0.05, paired t test). **(B)** The mRNA levels of m6A-related enzymes in BC tissues and paired normal breast tissues from GEO datasets (GSE22820 and GSE86374). **(C)** Overlap of the differentially expressed genes of interest from TCGA dataset and the GEO datasets (GSE22820 and GSE86374). **(D)** Kaplan-Meier OS curves based on IGF2BP1, FTO and WTAP mRNA expression using the online bioinformatics tool based on TCGA. **(E)** RNAs isolated from MDA-MB-231, LM2, BM6, and 1833 cells were used in dot blot assays with an anti-m6A antibody. MB (methylene blue) served as a loading control. **(F)** Protein levels of m6A-related enzymes were measured in MDA-MB-231, LM2, BM6, and 1833 cells by western blotting. **(G)** Representative IHC staining images of the BC samples probed with an anti-IGF2BP1 antibody (scale bars=100 µm or 50 µm) (left panel). Calculated IHC staining indices of BC samples (right panel, n=11, p=0.0002, paired t test). **(H)** Kaplan-Meier DFS and OS analyses of IGF2BP1 expression in patients with BC (n=80, p<0.01, log-rank test). **(I)** Representative IHC staining images of the primary BC samples with or without metastasis probed with an anti-IGF2BP1 antibody (scale bars=50 µm) (left panel). Calculated IHC staining indices of BC samples (right panel, n=7, p=0.0031, unpaired t test). The data are represented as the means ± SEM. * p<0.05; ** p<0.01; *** p<0.001.

To identify the roles of m6A regulators in BC, we first screened the expression of key m6A writers (METTL3, METTL14, and WTAP), erasers (ALKBH5 and FTO), and readers (YTHDC1, YTHDC2, YTHDF1, YTHDF3, IGF2BP1, IGF2BP2, and IGF2BP3) in BC samples and the paired normal breast tissues using The Cancer Genome Atlas (TCGA) data. The mRNA expression levels of YTHDC2, YTHDF1, YTHDF3, IGF2BP1, and IGF2BP3 were significantly increased in tumour tissues compared with paired normal breast tissues, while the METTL14, WTAP, FTO, and IGF2BP2 expression levels were significantly decreased in tumour tissues (Figure 1A). Correspondingly, the mRNA expression levels of METTL3, ALKBH5, and YTHDC1 showed no significant difference between the paired normal and BC tissues (Figure S1A). In addition, we examined all of these genes of interest in the BC tissues and paired controls in GEO datasets (GSE22820 and GSE86374) (Figure 1B). By taking the intersection of these results, we found that the mRNA expression levels of IGF2BP1, WTAP and FTO showed significant differences between the paired normal and BC tissues in all three datasets (Figure 1C). Among these three genes, only IGF2BP1 mRNA expression was associated with poor overall survival (OS) and disease-free survival (DFS) of BC patients based on TCGA datasets (Figure 1D and Figure S1C).

Furthermore, to explore the function of m6A modification in the metastasis of BC, we first detected the RNA m6A levels in MDA-MB-231, LM2 (MDA-MB-231 lung metastatic derivative), BM6 (MDA-MB-231 bone metastatic derivative), and 1833 (MDA-MB-231 brain metastatic derivative) cells. The RNA m6A levels were markedly increased in LM2, BM6, and 1833 cells compared with that in MDA-MB-231 cells as determined by dot blot assay (Figure 1E). Then, we examined the mRNA and protein expression levels of the key m6A writers (METTL3, METTL14, and WTAP), erasers (ALKBH5 and FTO), and readers (YTHDC1, YTHDC2, YTHDF1, YTHDF3, IGF2BP1, IGF2BP2, and IGF2BP3) in the four cell lines. Interestingly, the protein expression of IGF2BP1 was significantly higher in LM2, BM6, and 1833 cells than in MDA-MB-231 cells, but this phenomenon did not occur at the mRNA level (Figure 1F and Figure S1B).

We further confirmed this finding in our clinical samples and found elevated levels of the IGF2BP1 protein in BC tissues compared with paired normal breast tissues by immunohistochemical (IHC) staining (n=11; Figure 1G). To further evaluate the prognostic effect of IGF2BP1, we also assessed IGF2BP1 expression by IHC staining in 80 BC tissue samples. BC patients with higher IGF2BP1 expression had shorter OS and DFS (Figure 1H and Figure S1D). Moreover, primary BC tissues from patients with distant metastasis had higher expression of IGF2BP1 than those without distant metastases (n=7; Figure 1I). These data suggest that IGF2BP1 might play a crucial role in the malignant process of BC, especially in its metastasis.

### IGF2BP1 promoted BC metastasis *in vitro* and *in vivo*

**Figure 2:**
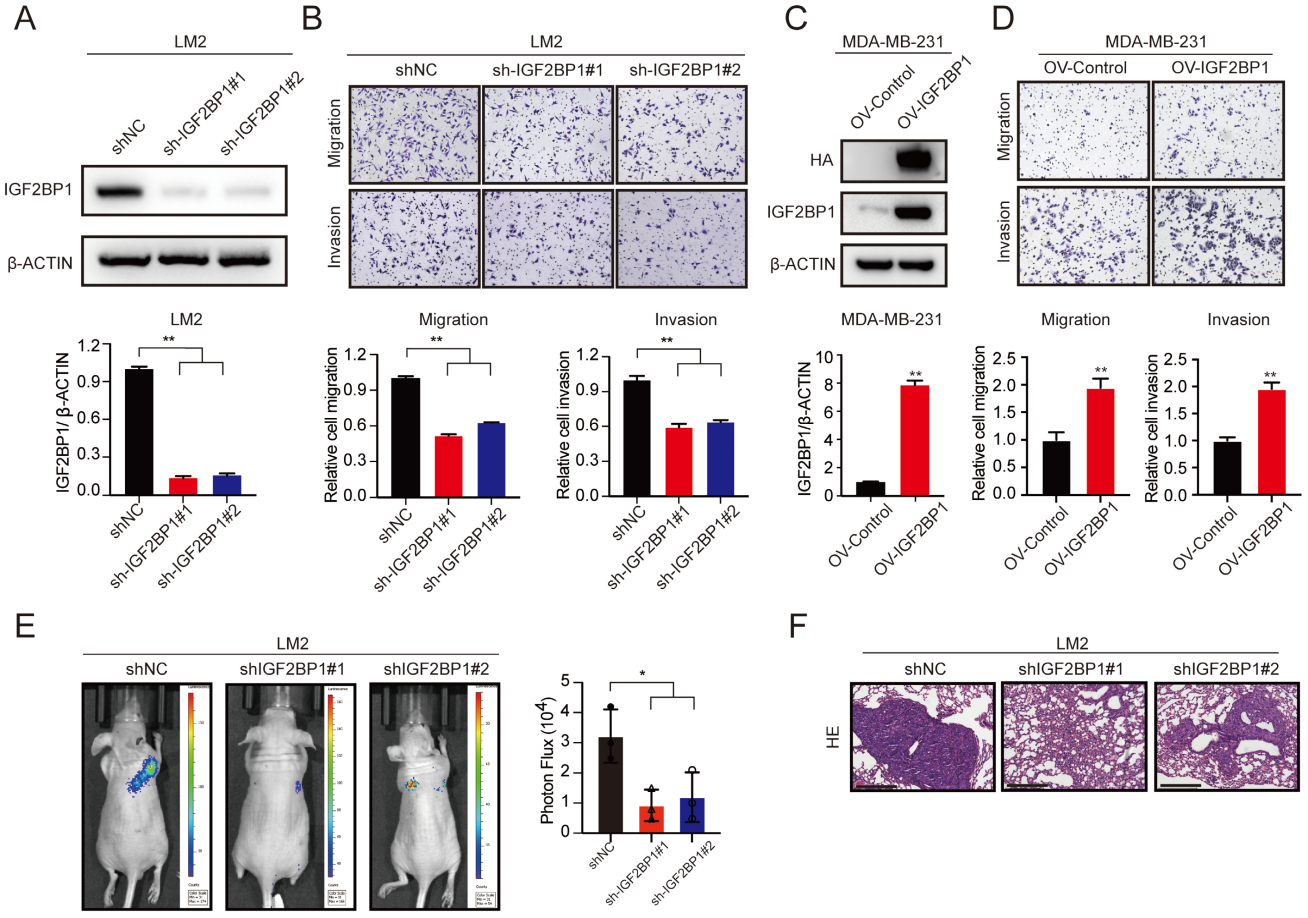
** IGF2BP1 promoted breast cancer metastasis *in vitro* and *in vivo*. (A)** The protein level of IGF2BP1 in LM2 cells with stable downregulation of IGF2BP1 measured by western blotting (upper panel). The grey values of the images were calculated using ImageJ (bottom panel). **(B)** Cell migration and invasion assays of LM2 cells deficient in IGF2BP1. Representative images and quantification of the cell migration and invasion assays are shown. **(C)** The protein levels of HA-tag and IGF2BP1 in MDA-MB-231 cells overexpressing IGF2BP1 measured by western blotting (upper panel). The grey values of the images were calculated using ImageJ (bottom panel). **(D)** Migration and invasion of MDA-MB-231 cells overexpressing IGF2BP1. Representative images and quantification of cell migration and invasion are shown. **(E-F)** IGF2BP1 downregulation led to markedly decreased BC lung metastasis in female nude mice (total n=9). Representative bioluminescence images and quantification of bioluminescence (E) of mice and HE staining (scale bars=275 µm) (F) of pulmonary metastases 8 weeks after tail vein injection of IGF2BP1 downregulated or vector control LM2 cells. The data are represented as the means ± SEM. * p<0.05; ** p<0.01; *** p<0.001.

To determine the role of IGF2BP1 in BC metastasis, we first established stable IGF2BP1 knockdown BC cells (LM2 and 1833) (Figure 2A and Figure S2A). The results showed that knockdown of IGF2BP1 suppressed the migration and invasion ability of LM2 and 1833 cells (Figure 2B and Figure S2B-C). In addition, we constructed IGF2BP1-overexpressing BC cells using the parental MDA-MB-231 cell line (Figure 2C). The results showed that ectopic expression of IGF2BP1 significantly promoted the migration and invasion ability of MDA-MB-231 cells (Figure 2D).

We further explored the prometastatic effect of IGF2BP1 *in vivo*. LM2-luciferase cells with IGF2BP1 knockdown and the corresponding control cells were injected into nude mice through the tail vein. Intriguingly, downregulation of IGF2BP1 significantly suppressed lung metastasis compared with that in the control group after 4 weeks, as indicated by bioluminescence imaging and haematoxylin and eosin (HE) staining of pulmonary metastases (Figure 2E-F). These data suggest that IGF2BP1 may act as an oncogene that promotes BC metastasis.

### USP10 bound to, deubiquitinated, and stabilized the IGF2BP1 protein

**Figure 3:**
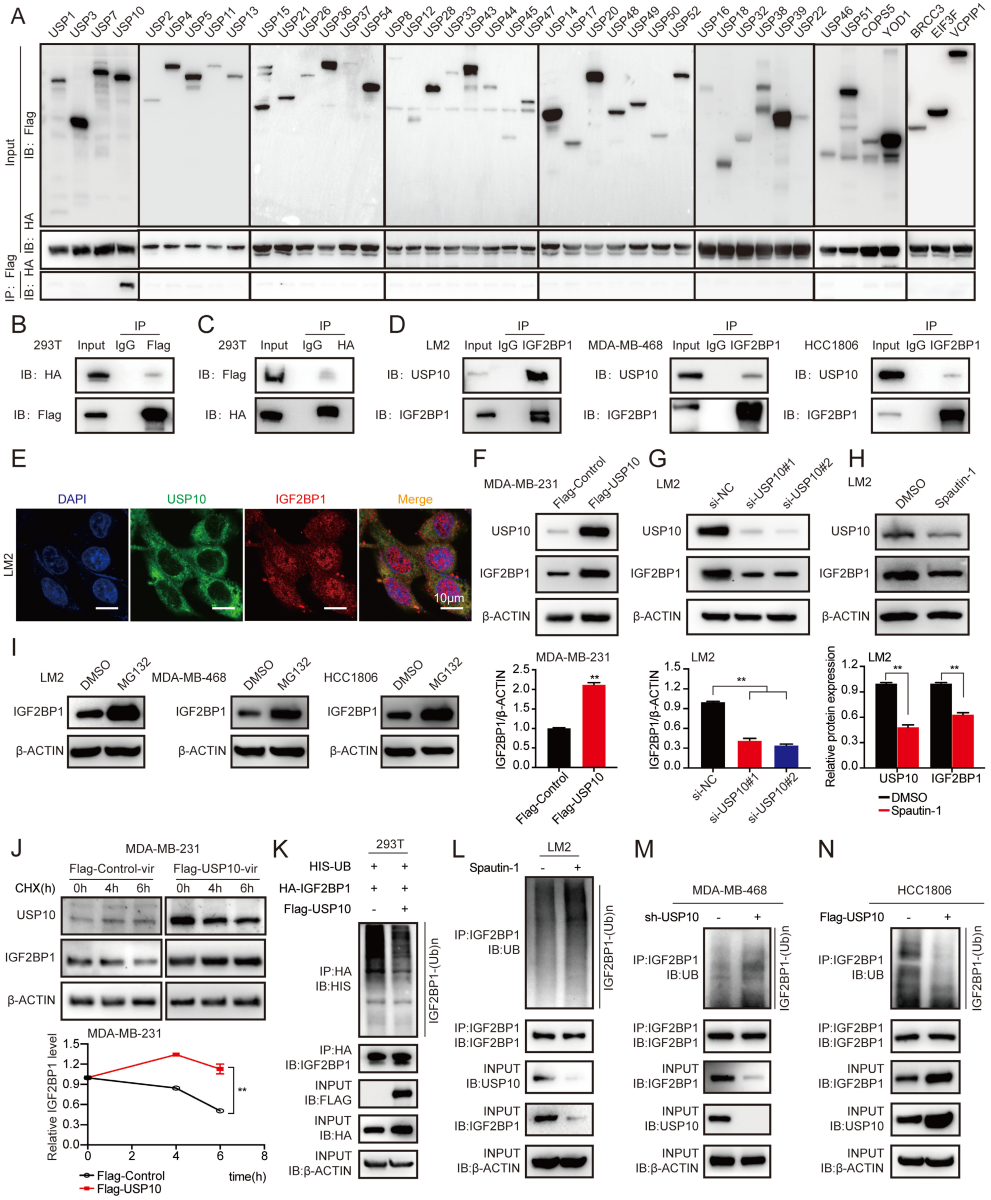
** USP10 bound to and stabilized IGF2BP1 by deubiquitination. (A-C)** USP10 specifically bound to IGF2BP1. HEK293T cells were transfected with FLAG-tagged DUBs and HA-tagged IGF2BP1 plasmids for 72 h, immunoprecipitated with FLAG or HA beads along with IgG control beads and then subjected to immunoblotting (IB) with the indicated antibodies. **(D)** Endogenous USP10 bound to IGF2BP1 in LM2 cells, MDA-MB-468 cells and HCC1806 cells. Cell lysates from LM2 cells, MDA-MB-468 cells and HCC1806 cells were immunoprecipitated with an anti-IGF2BP1 antibody along with a normal IgG control, followed by IB with the indicated antibodies. **(E)** Immunofluorescence (IF) assays were used to detect the expression and location of USP10 and IGF2BP1 in LM2 cells (scale bars=10 µm). **(F-G)** IGF2BP1 protein levels were detected by overexpressing or downregulating USP10 in MDA-MB-231 and LM2 cells. Cells were transfected with USP10 plasmids or siRNAs targeting USP10 for 48 h (upper panel). Densitometry quantification was carried out by ImageJ (bottom panel). **(H)** LM2 cells were treated with 10 μM Spautin-1 for 6 h. The protein levels of USP10 and IGF2BP1 were measured by western blotting (upper panel). Grey value quantification was carried out by ImageJ (bottom panel). **(I)** LM2 cells, MDA-MB-468 cells and HCC1806 cells were treated with 10 μM MG132 for 6 h, and the protein levels of IGF2BP1 were measured by western blotting. **(J)** USP10-overexpressing MDA-MB-231 cells and control cells were treated with 100 μg/ml CHX for the indicated time periods. The protein levels of IGF2BP1 were measured by western blotting (upper panel). The decay curves are shown based on quantification of the grey values by ImageJ (bottom panel). **(K)** HEK293T cells were transfected with Flag-tagged USP10, Flag-tagged NC, HA-tagged IGF2BP1, and HIS-tagged UB plasmids for 72 h, followed by treatment with 10 μM MG132 for 6 h. IGF2BP1 protein was pulled down by HA beads and then subjected to IB with the indicated Abs. **(L)** LM2 cells were treated with 10 μM Spautin-1 for 6 h, followed by 10 μM MG132 for 6 h. IGF2BP1 protein was pulled down and then subjected to IB with the indicated Abs. **(M-N)** USP10 was overexpressed or downregulated in MDA-MB-468 cells and HCC1806 cells followed by treatment with 10 μM MG132 for 6 h. IGF2BP1 protein was pulled down and then subjected to IB with the indicated Abs. The data are represented as the means ± SEM. * p<0.05; ** p<0.01; *** p<0.001.

As shown in Figure 1F and Figure S1B, the IGF2BP1 protein level, but not the mRNA level, was higher in LM2 and 1833 cells than in MDA-MB-231 cells; therefore, we speculated that the IGF2BP1 protein may be more stable in metastatic BC cells. It has been reported that TRIM25, an E3 ubiquitin ligase, can regulate IGF2BP1 ubiquitination and degradation 33. However, the deubiquitinase of IGF2BP1 is still unknown. To this end, we screened a panel of 43 DUBs (USP1, USP2, USP3, USP4, USP5, USP7, USP8, USP10, USP11, USP12, USP13, USP14, USP15, USP16, USP17, USP18, USP20, USP21, USP22, USP26, USP28, USP32, USP33, USP36, USP37, USP38, USP39, USP43, USP44, USP45, USP46, USP47, USP48, USP49, USP50, USP51, USP52, USP54, COPS5, YOD1, BRCC3, EIF3F, and VCPIP1) to investigate the potential interaction between each DUB and IGF2BP1 by coimmunoprecipitation (co-IP) assays. We found that USP10 clearly bound to IGF2BP1 (Figure 3A). Furthermore, we discovered that exogenous USP10 and IGF2BP1 bound to each other in HEK293T cells using co-IP assays (Figure 3B-C), and endogenous USP10 and IGF2BP1 could interact in LM2 cells, MDA-MB-468 cells and HCC1806 cells (Figure 3D). In addition, as shown in Figure 3E, USP10 and IGF2BP1 were mainly colocalized in the cytoplasm. These data suggest that USP10 may function as the deubiquitinase of IGF2BP1.

To determine whether USP10 could affect IGF2BP1 protein levels, USP10-overexpressing plasmids and their specific siRNAs were transfected into MDA-MB-231, LM2, and 1833 cells. Overexpression of USP10 elevated the expression of IGF2BP1 in MDA-MB-231 cells, while knockdown of USP10 reduced the protein level of IGF2BP1 in LM2 and 1833 cells (Figure 3F-G and Figure S3A-B). Additionally, IGF2BP1 protein expression was significantly decreased in LM2 cells treated with Spautin-1, a specific USP10 inhibitor 40 (Figure 3H). Meanwhile, we also examined the basal IGF2BP1 and USP10 expressions in MDA-MB-231, LM2, BM6, 1833, MDA-MB-468 and HCC1806 cells (figure S3C). Moreover, the IGF2BP1 protein level was increased in LM2 cells, MDA-MB-468 cells and HCC1806 cells treated with MG132, a proteasome inhibitor 41 (Figure 3I). Consistently, ectopic expression of USP10 prolonged the half-life of the IGF2BP1 protein in MDA-MB-231 cells treated with cycloheximide (CHX), a protein synthesis inhibitor 42 (Figure 3J). Furthermore, overexpression of USP10 decreased the level of ubiquitinated IGF2BP1 in LM2 cells compared with the corresponding control cells (Figure 3K). Inhibiting USP10 with Spautin-1 increased the level of ubiquitinated IGF2BP1 in LM2 cells compared with the corresponding control cells (Figure 3L). Moreover, we found that the level of ubiquitinated IGF2BP1 was enhanced or reduced in MDA-MB-468 cells and HCC1806 cells after USP10 knockdown or overexpression (Figure 3M-N and Figure S3D). Collectively, these results illustrated that the deubiquitinase USP10 interacted with IGF2BP1 and stabilized it at the protein level in BC cells.

### The USP10/IGF2BP1 axis promoted BC metastasis *in vitro* and *in vivo*

**Figure 4:**
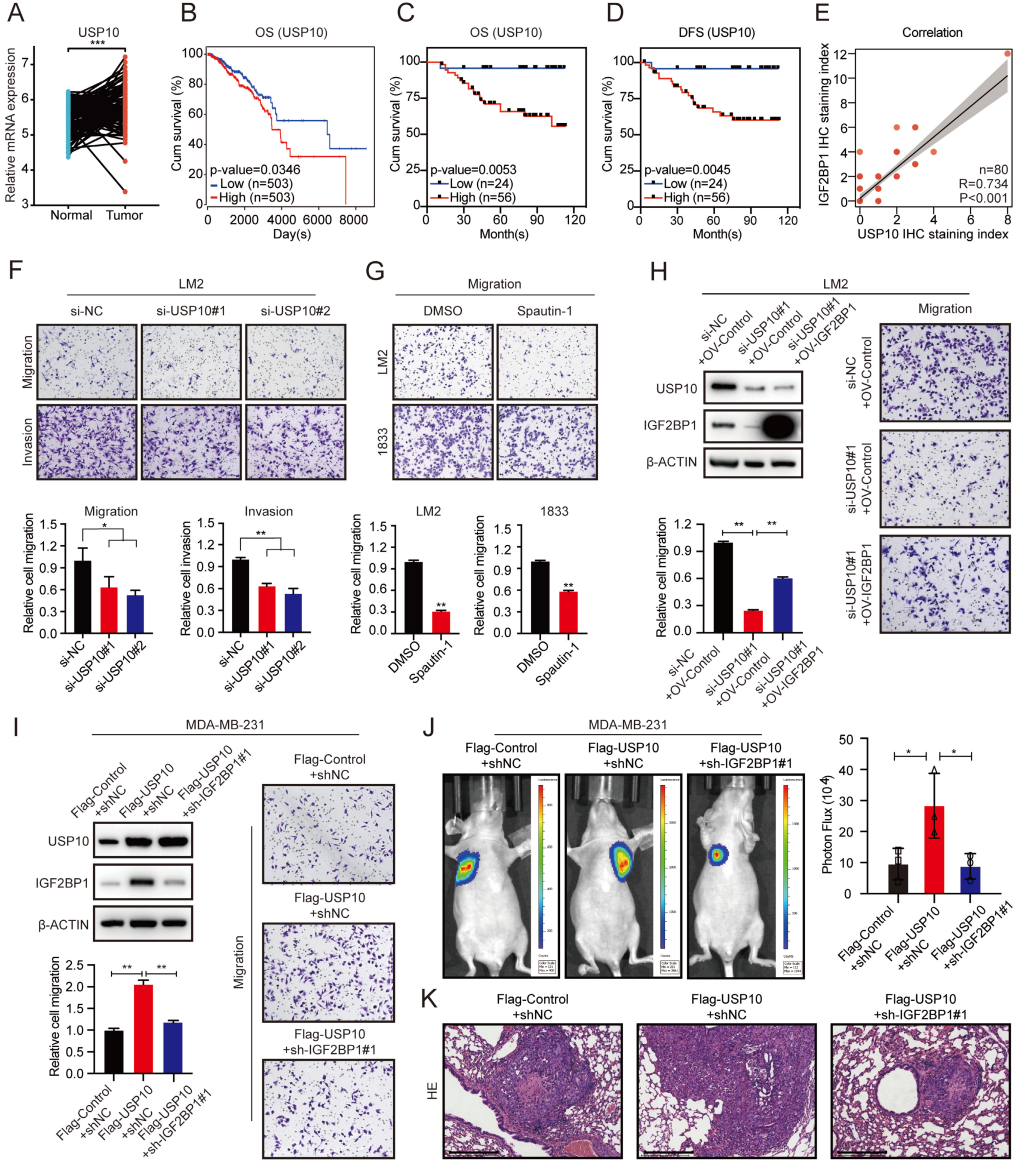
** The USP10/IGF2BP1 axis promoted BC metastasis *in vitro* and *in vivo.* (A)** USP10 mRNA expression in 112 BC tissues and paired normal breast tissues was analysed using TCGA data (n=112, p<0.001, paired t test). **(B)** Kaplan-Meier OS curves were analysed based on USP10 mRNA expression from TCGA data using the online bioinformatics tool. **(C-D)** Kaplan-Meier OS and DFS analyses of IGF2BP1 expression in patients with BC were performed (n=80, p<0.01, log-rank test). **(E)** The correlation between IGF2BP1 expression and USP10 expression in BC patients was analysed (n=80, p<0.001, Spearman test). **(F-G)** USP10 knockdown using targeting siRNAs or Spautin-1 effectively inhibited the migration and invasion abilities of LM2 cells or the migration ability of both LM2 and 1833 cells (upper panel). A graphical representation of the quantified data is shown (bottom panel). **(H)** IGF2BP1 overexpression rescued the reduced migration ability of LM2 cells caused by siRNAs targeting USP10. Representative images of the migration assays (right panel). Western blotting images and corresponding quantification of the migration assay (left panel). **(I)** Knockdown of IGF2BP1 inhibited the improved migration ability of MDA-MB-231 cells caused by USP10 overexpression. Representative images of migration assays (right panel). Western blotting and the corresponding quantification of migration ability results (left panel). **(J)** Downregulating IGF2BP1 blocked the enhanced metastatic ability of MDA-MB-231 cells induced by USP10 overexpression in female nude mice (total n=9). Representative bioluminescence images of mice 8 weeks after tail vein injection of treated MDA-MB-231 cells attached to the lung region and the corresponding quantification. **(K)** Corresponding HE staining images of pulmonary metastases (scale bars=275 µm). The data are represented as the means ± SEM. * p<0.05; ** p<0.01; *** p<0.001.

The expression of USP10 was significantly upregulated in BC tissues compared with paired normal breast tissues based on TCGA data (Figure 4A). Moreover, higher expression of USP10 was associated with a poor prognosis of BC patients (Figure 4B). We performed IHC staining of 80 BC tissues and confirmed that higher USP10 expression was associated with shorter OS and DFS in BC patients (Figure 4C-D and Figure S4A). As shown in Figure S4B, there was no significant relationship between the mRNA expression of USP10 and IGF2BP1. However, the protein level of USP10 were significantly correlated with that of IGF2BP1 in BC samples (n=80, p<0.001, Figure 4E).

Subsequently, knocking down USP10 dramatically suppressed the migration and invasion of LM2 and 1833 cells (Figure 4F and Figure S4C-D). The USP10 inhibitor Spautin-1 also inhibited the migration of LM2 and 1833 cells (Figure 4G). Moreover, overexpressing IGF2BP1 markedly rescued the inhibition of migration caused by USP10 knockdown in LM2 and 1833 cells (Figure 4H and Figure S4E-F). Conversely, suppressing IGF2BP1 significantly blocked USP10 overexpression-induced migration in MDA-MB-231 cells (Figure 4I). Next, BC lung metastasis mouse models were constructed, and the number and size of lung metastatic lesions were analysed by bioluminescence imaging and HE staining of the pulmonary metastases at 8 weeks after injection. The results indicated that IGF2BP1 could block BC metastasis caused by USP10 overexpression *in vivo* (Figure 4J-K). These results suggest a pivotal role of the USP10/IGF2BP1 axis in BC metastasis.

### IGF2BP1 recognized the m6A modification on CPT1A mRNA to maintain mRNA stability

**Figure 5:**
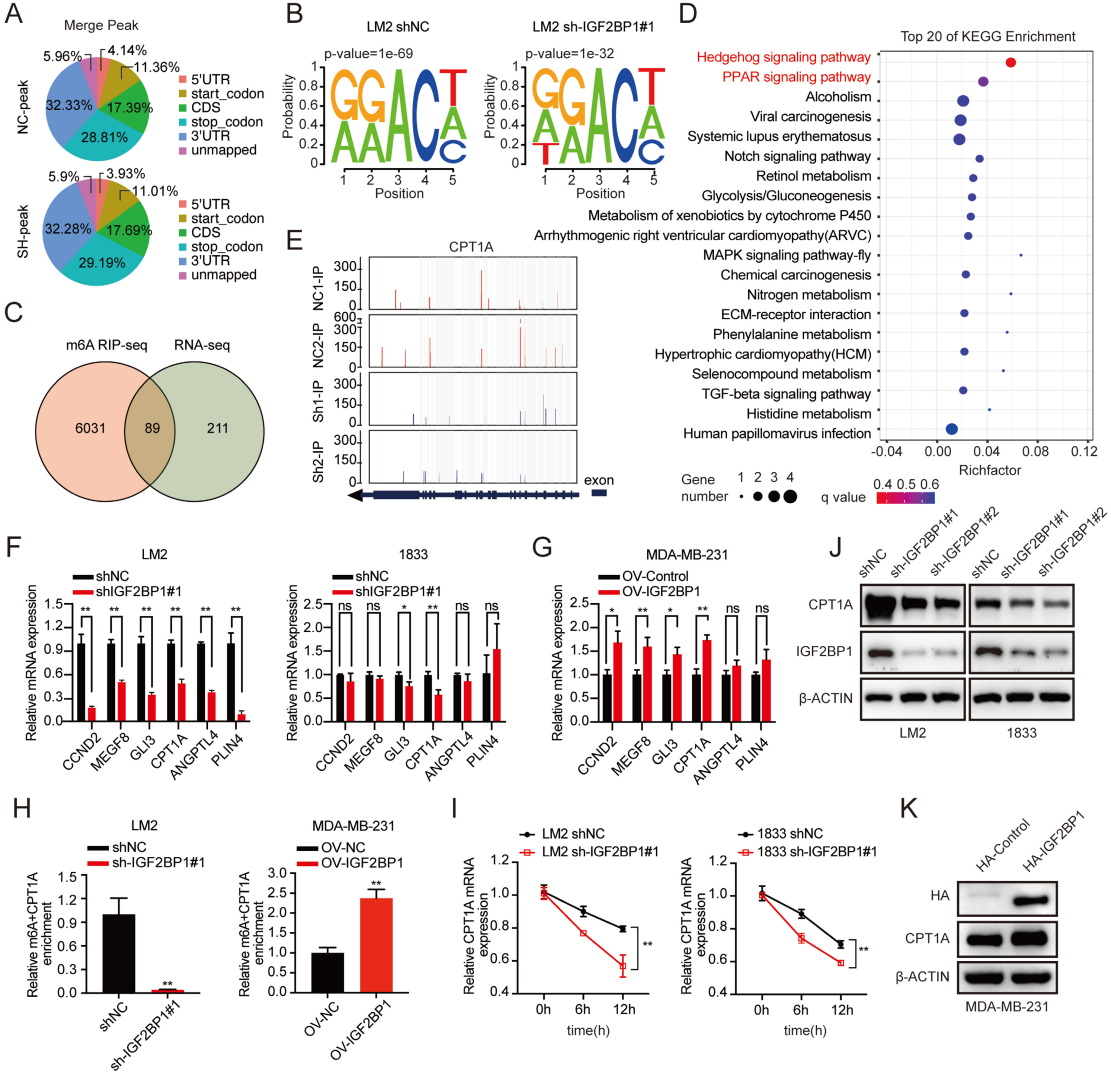
** IGF2BP1 stabilized CPT1A via m6A modification. (A)** The distribution of m6A peaks in each transcript segment of IGF2BP1 knockdown LM2 cells and vector control LM2 cells was analysed. **(B)** Enrichment analyses for an averaged base frequency matrix based on the frequency distribution of a specific motif (RRACH) in IGF2BP1 knockdown and control LM2 cells performed by MeRIP-seq. **(C)** The overlapping differentially expressed genes determined by RNA-seq and MeRIP-seq performed using LM2 cells with stable knockdown of IGF2BP1 and compared with their corresponding controls. **(D)** The top 20 enriched KEGG pathways based on the overlapping genes in (C). **(E)** The m6A abundances detected by MeRIP-seq on CPT1A mRNA transcripts in IGF2BP1 knockdown LM2 cells and the corresponding control cells plotted by Integrative Genomics Viewer (IGV). **(F-G)** The mRNA expression levels of six candidate genes were examined in IGF2BP1 knockdown LM2/1833 cells (F) and IGF2BP1-overexpressing MDA-MB-231 cells (G) and compared with the corresponding control cells by qRT-PCR. **(H)** MeRIP-qPCR analysis was employed to demonstrate the decrease or increase in m6A modifications on CPT1A mRNA caused by IGF2BP1 downregulation and overexpression in LM2/MDA-MB-231 cells. **(I)** The mRNA expression of CPT1A was detected in IGF2BP1 knockdown LM2/1833 cells compared with the corresponding control LM2/1833 cells treated with actinomycin D (2 µg/mL) at the indicated time points by qRT-PCR. **(J-K)** The protein expression of CPT1A was examined in IGF2BP1 knockdown LM2 cells and 1833 cells and IGF2BP1-overexpressing MDA-MB-231 cells and compared with the corresponding control cells. The data are represented as the means ± SEM. * p<0.05; ** p<0.01; *** p<0.001.

To explore the molecular mechanism by which IGF2BP1 promoted BC metastasis, we performed methylated RNA immunoprecipitation sequencing (MeRIP-seq) and RNA sequencing (RNA-seq) in LM2 cells with stable IGF2BP1 knockdown and the corresponding control cells. The m6A peaks identified by MeRIP-seq were mainly distributed in the coding sequence (CDS), 3' untranslated region (3'UTR), start codon, and stop codon (Figure 5A). m6A occurs mostly in the RRACH (R=G or A, H=A, C, or U) consensus sequence 20, 43. Our MeRIP-seq results revealed that the GGACT motif was highly enriched in the immunopurified RNA in both control LM2 cells and IGF2BP1 knockdown LM2 cells and the abundance of m6A was decreased in the mRNA of IGF2BP1 knockdown LM2 cells (Figure 5B). Furthermore, MeRIP-seq revealed that 6120 transcripts had fewer m6A peaks in IGF2BP1 knockdown cells than in control cells (fold change >1.5). Moreover, RNA-seq revealed that 300 transcripts were markedly decreased in IGF2BP1 knockdown cells compared with control cells (fold change >1.5) (Figure 5C and Figure S5A-B). Integrating the MeRIP-seq and RNA-seq results, 89 transcripts overlapped (Figure 5C). Furthermore, Kyoto Encyclopedia of Genes and Genomes (KEGG) enrichment analysis showed that these transcripts mainly functioned in the Hedgehog signaling pathway and PPAR signaling pathway (Figure 5D). Then, the expression of six candidate genes (CCND2, MEGF8, GLI3, CPT1A, ANGPTL4, PLIN4) in these two pathways was further verified in IGF2BP1-deficient LM2/1833 cells and IGF2BP1-overexpressing MDA-MB-231 cells by qRT-PCR (Figure 5F-G and Figure S5C). We found that the expression of CPT1A and GLI3 was consistently regulated by IGF2BP1 in these cells. Additionally, the m6A abundance in CPT1A and GLI3 mRNA was significantly decreased upon IGF2BP1 knockdown, as shown by the MeRIP-qPCR assay (Figure 5H and Figure S5D). Overexpression of IGF2BP1 also enriched the m6A abundance in CPT1A mRNA (Figure 5H). The Integrative Genomics Viewer (IGV) also indicated that the m6A levels on the exons of CPT1A mRNA were decreased in IGF2BP1 knockdown LM2 cells compared with control cells (Figure 5E). Moreover, the protein level of CPT1A was decreased in IGF2BP1 knockdown LM2/1833 cells but increased in IGF2BP1-overexpressing MDA-MB-231 cells compared with the corresponding control cells (Figure 5J-K). IGF2BP1 is recognized to stabilize target mRNA transcripts with m6A motifs 23. We then treated IGF2BP1 knockdown LM2 cells and control cells with actinomycin D, an inhibitor of transcription 44, which showed that the mRNA level of CPT1A was less stable in IGF2BP1 knockdown LM2/1833 cells (Figure 5I). However, we found that knockdown of IGF2BP1 reduced m6A modification of GLI3, but did not affect GLI3 mRNA stability, indicating that IGF2BP1 may not completely depend on m6A modification to regulate GLI3 expression (Figure S5D-E). Together, our findings indicated that m6A modification maintains stable CPT1A expression in an IGF2BP1-dependent manner in metastatic BC cells.

### IGF2BP1 promoted BC metastasis via the upregulation of CPT1A

**Figure 6:**
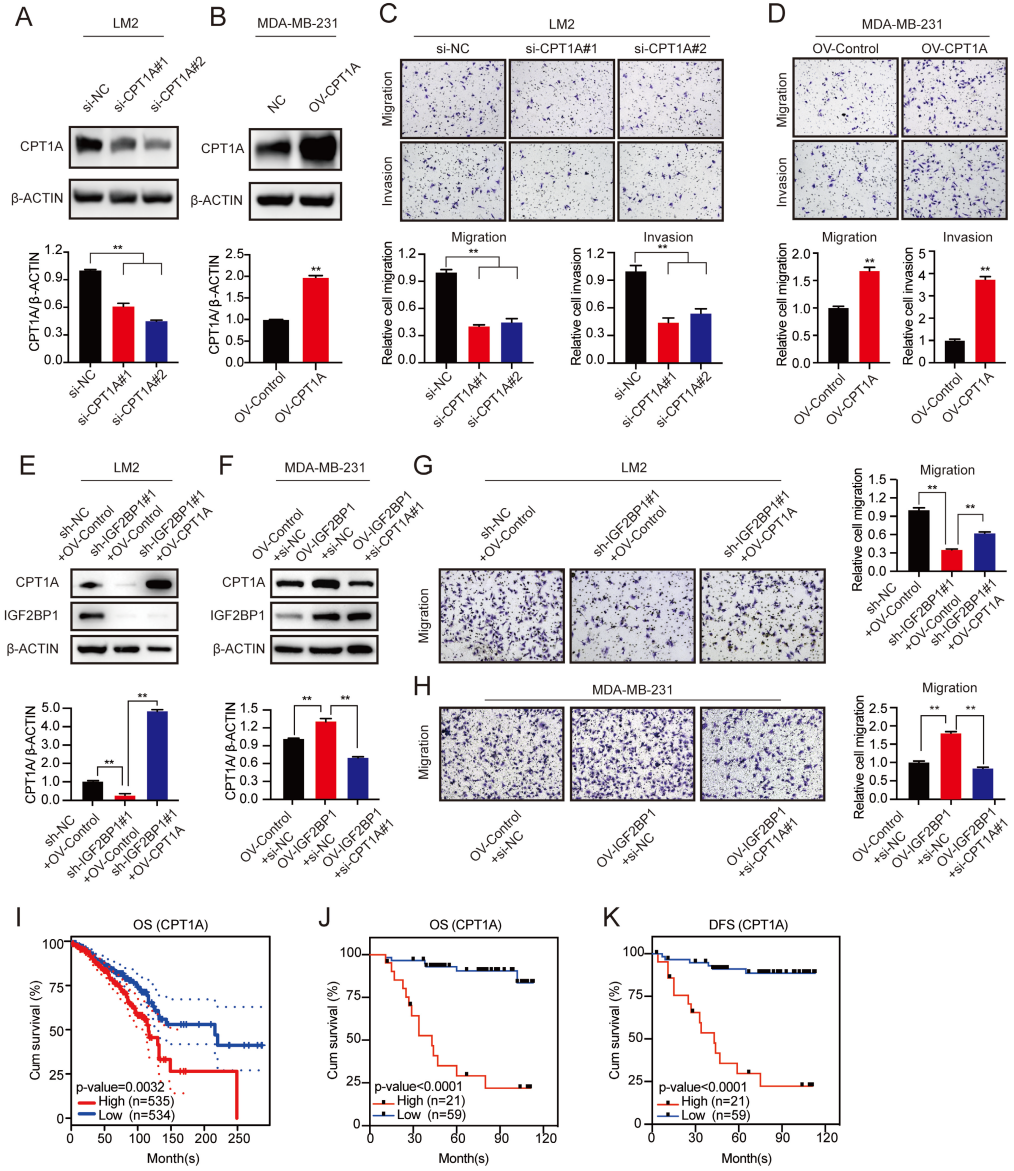
** IGF2BP1 promoted BC metastasis by upregulating CPT1A. (A-B)** CPT1A knockdown and overexpression efficiency verified by western blotting in LM2 cells (A) and MDA-MB-231 cells (B) (upper panel), and the corresponding quantification of the grey values (bottom panel). **(C-D)** Migration and invasion of LM2 cells with CPT1A knockdown (C) or MDA-MB-231 cells with CPT1A overexpression (D). Representative images (upper panel) and quantification (bottom panel) of cell migration and invasion are shown. **(E&G)** Upregulating CPT1A repaired the decreased migration ability of LM2 cells with stable knockdown of IGF2BP1 caused by targeting lentiviruses. Western blotting images and corresponding quantification analysis are shown (E). Representative images of migration assays and the corresponding quantification (G). **(F&H)** Knocking down CPT1A blocked the increased migration ability in IGF2BP1-overexpressing LM2 cells. The results of western blotting and corresponding quantification are shown (F). Representative migration assay results and the corresponding quantification are shown on the right (H). **(I)** Kaplan-Meier OS curves were analysed based on CPT1A mRNA expression using the online bioinformatics tool. **(J-K)** Kaplan-Meier OS and DFS analyses were carried out based on the CPT1A IHC staining indices in patients with BC (n=80, p<0.0001, log-rank test). The data are represented as the means ± SEM. * p<0.05; ** p<0.01; *** p<0.001.

To further characterize the function of CPT1A in BC metastasis, we first downregulated CPT1A with two different specific siRNAs and confirmed the knockdown efficiency by western blotting (Figure 6A and Figure S6A). In addition, MDA-MB-231 cells stably overexpressing CPT1A were constructed (Figure 6B). We found that knockdown of CPT1A dramatically suppressed the migration and invasion abilities of LM2 and 1833 cells, while CPT1A overexpression promoted the migration and invasion abilities of MDA-MB-231 cells (Figure 6C-D and Figure S6B). Furthermore, ectopic expression of CPT1A rescued the decreased migration ability of IGF2BP1 knockdown LM2 and 1833 cells (Figure 6E&G and Figure S6C). Moreover, downregulating CPT1A by specific siRNAs blocked the enhanced migration abilities observed by overexpression of IGF2BP1 (Figure 6F&H). Thus, our data suggest that IGF2BP1 promoted BC metastasis by upregulating CPT1A expression.

Moreover, high expression of CPT1A mRNA also predicted poor OS of patients according to TCGA data (Figure 6I). Furthermore, IHC staining in 80 clinical BC samples confirmed that patients with higher CPT1A protein expression had shorter OS and DFS (Figure 6J-K and Figure S6D).

**Figure 7:**
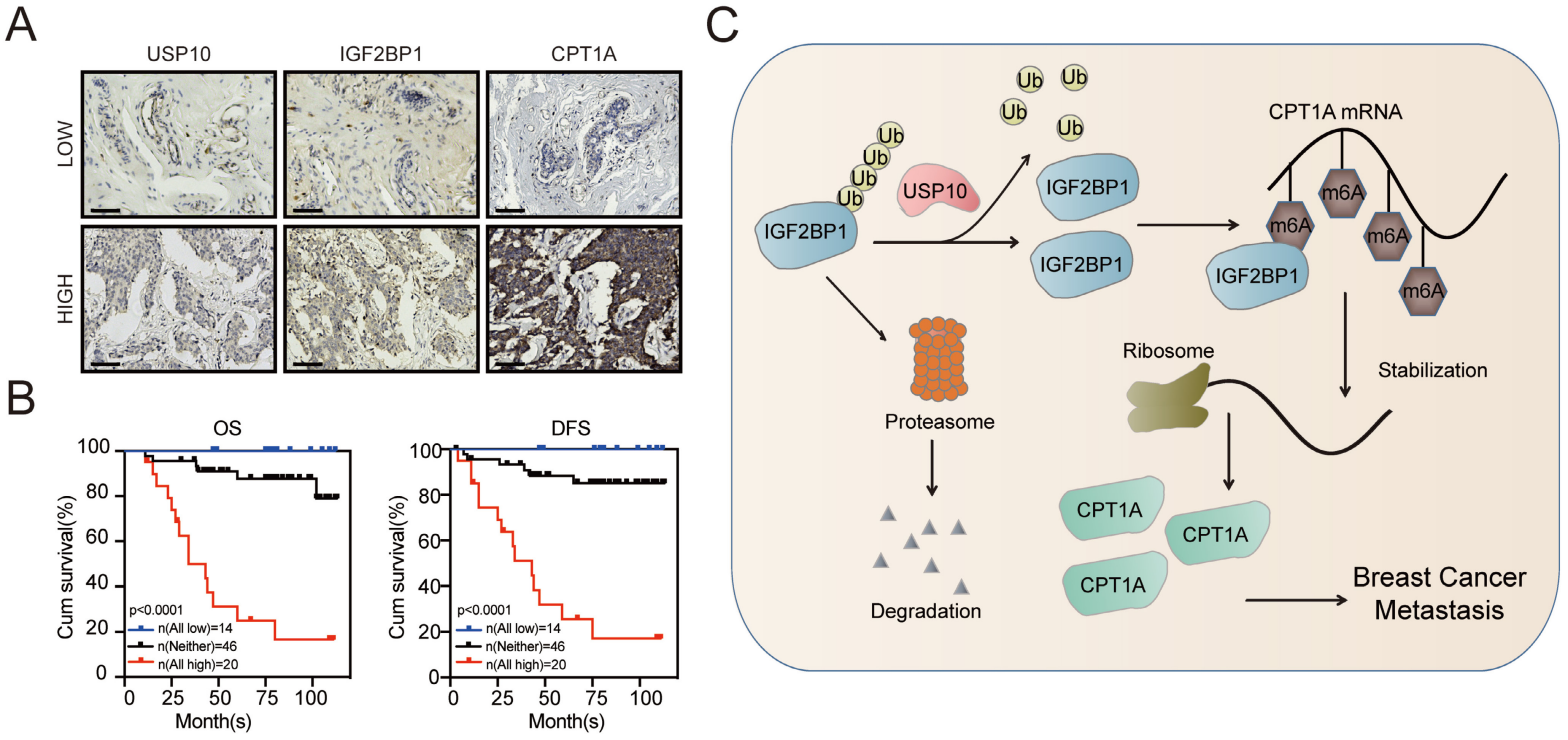
** The clinical significance of the USP10/IGF2BP1/CPT1A axis in BC prognosis. (A)** Representative IHC staining images of USP10, IGF2BP1, and CPT1A in the cancerous tissues of BC patients with high or low USP10 levels. **(B)** BC patients with high expression of USP10, IGF2BP1, and CPT1A had the lowest OS and DFS compared with the groups with low and no expression of these indicators. Kaplan-Meier OS and DFS analyses of 80 BC patients were performed (n=80, p<0.01, log-rank test). **(C)** The graphic illustrates that the USP10/IGF2BP1/CPT1A axis promoted BC metastasis.

To further confirm the role of the USP10/IGF2BP1/CPT1A axis in BC metastasis, we analysed the IHC staining images of USP10, IGF2BP1, and CPT1A in our BC samples. As shown in Figure 7A, the USP10 levels distinctly correlated with the IGF2BP1 and CPT1A levels in BC tissues. Moreover, BC patients with high levels of USP10, IGF2BP1, and CPT1A had the worst outcomes compared with those with low levels of ang or all of these indicators. (Figure 7B). These data suggest that the USP10/IGF2BP1/CPT1A axis may boost human BC progression and is correlated with BC patient survival.

## Discussion

The decisive role of m6A modification in cancer progression has been widely reported in recent years 19, 45. m6A modification is dynamically regulated by its writers, erasers, and readers, which are involved in the progression of cancer metastasis 13, 16, 46, including BC 15, 47-50. Here, we found that the m6A reader IGF2BP1 was upregulated in BC tissues, especially BC metastatic lesions, and was associated with a poor prognosis of BC. Functionally, we first identified that the deubiquitinase USP10 could bind to and stabilize the IGF2BP1 protein, resulting in its high expression level in BC. Furthermore, IGF2BP1 directly bound to and recognized the m6A modification on CPT1A mRNA to maintain its stability, which had been reported to endow BC cells with the potential for metastasis 35, 37. Thus, IGF2BP1 is capable of promoting BC distant metastasis (Figure 7C).

IGF2BP1 is regarded as a classic RNA-binding protein involved in the regulation of gene expression 51, 52. Recent evidence has confirmed that IGF2BP1 can function as an m6A reader to recognize m6A sites and enhance targeted mRNA stability 23. Recently, IGF2BP1 was reported to promote BC lung metastasis by cooperating with METTL3 and FTO and stabilizing the keratin 7 (KRT7)-AS/KRT7 mRNA duplex by recognizing m6A at A877 on KRT7-AS 27. Here, we first discovered that IGF2BP1 was upregulated in BC tissues, especially in BC patients with distant metastasis. Moreover, the IGF2BP1 protein level, but not its mRNA level, was consistently elevated in the lung, bone, and brain BC metastatic derivative cell lines compared with the parental cell line, suggesting that IGF2BP1 plays an extensive role in the distant metastasis of BC.

The discrepancies of IGF2BP1 function in breast cancers need to be noted. It has been reported that IGF2BP1 functions as a tumor suppressor 53, 54. However, recent studies from different research groups demonstrated the oncogenic roles of IGF2BP1 in breast cancer, maintaining the glycolysis and stemness of BCSCs through IGF2BP1/c-Myc axis 55, 56, promoting proliferation of breast cancer cells 57 and contributing the progression of breast cancer 58. Based on GEO and TCGA datasets, Zhong et al. found that IGF2BP1 was an independent prognostic factor of breast cancer, and higher expression level of IGF2BP1 was associated with shorter overall survival of breast cancer patients 59, which was consistent with our results. In our study, we found the oncogenic role of IGF2BP1 in TNBC cell lines, and we also showed that high expression of IGF2BP1 was clinically correlated with metastasis in breast cancer patients. Therefore, due to the complexity of the pathological types of breast cancer, more studies are warranted.

The IGF2BP1 protein level has been reported to be widely upregulated in several aggressive cancers 27, 60-64. However, the modulatory mechanisms underlying the abnormally elevated protein levels of IGF2BP1 in cancers are poorly understood. Previous studies have shown that posttranslational modification of some m6A regulators, such as ubiquitination 65, phosphorylation 66, sumoylation 67, 68, and lactylation 69, 70, could influence their biological function 33, 65, 71. For example, F-box and WD repeat domain-containing 7 (FBW7), an E3-ubiquitin ligase, ubiquitinates YTHDF2 and suppresses tumour progression by antagonizing the tumour-promoting effect of YTHDF2 in ovarian cancer 72. The ubiquitination of IGF2BP1/2/3 was first reported in their interaction with the E3-ubiquitin ligase tripartite motif-containing protein 25 (TRIM25) in non-small cell lung cancer (NSCLC) 33. In addition, F-box/SPRY domain-containing protein 1 (FBXO45), an E3-ubiquitin ligase, was found to promote IGF2BP1 ubiquitination and attenuate hepatocellular carcinoma (HCC) progression 73. However, the deubiquitinase of IGF2BP1 has not yet been reported. Our study first identified USP10 as the deubiquitinating enzyme of IGF2BP1, which maintained a high level of IGF2BP1 expression in BC. Moreover, we highlighted that USP10 was an independent prognostic marker that predicts the outcomes of BC patients.

Metabolic reprogramming is a hallmark of malignant tumours 74. Fatty acid oxidation (FAO) is of great importance in tumour progression because it increases the production of ATP and NADPH and enables cancer cells to survive under metabolic stress 75, 76. In our previous study on oesophageal cancer (ESCA), the m6A reader HNRNPA2B1 upregulated the expression of ATP citrate lyase (ACLY) and acetyl-CoA carboxylase (ACC1), two fatty acid synthetic enzymes, and promoted ESCA progression and metastasis 77. In this study, by MeRIP and experimental verification, we first found that the FAO rate-limiting enzyme CPT1A 75, 78-80 was the downstream target of IGF2BP1. In colorectal cancer (CRC), CPT1A has been shown to strengthen resistance to anoikis and eliminate reactive oxygen species (ROS), thus promoting CRC cell metastatic capacity 79. Here, we found that CPT1A mRNA could be stabilized by IGF2BP1 via recognition of its m6A modification, which led to its high level in BC and mediated IGF2BP1-induced distant metastasis in BC.

## Conclusion

Our work revealed that the USP10/IGF2BP1/CPT1A axis promoted BC metastasis. In clinical samples, the USP10 levels were correlated with the IGF2BP1 and CPT1A levels in BC tissues, and BC patients with high levels of USP10, IGF2BP1, and CPT1A had the worst outcome. Therefore, the USP10/IGF2BP1/CPT1A axis might be a potential predictor and therapeutic target for BC.

## Supplementary Material

Supplementary figures and tables.

## Abbreviations

m6A: N6-Methyladenosine
IGF2BP1: IGF2 mRNA binding protein 1
USP: ubiquitin-specific protease
CPT1A: carnitine palmitoyl transferase 1 A
FAO: fatty acid oxidation
BC: breast cancer
GC: gastrointestinal cancer
OC: ovarian cancer
HCC: hepatocellular carcinoma
METTL3/14/16: methyltransferase-like 3/14/16
WTAP: Wilms tumor 1-associated protein
RBM15/15B: RNA-binding motif protein 15/15B
VIRMA: also called KIAA1429, vir-like m6A methyltransferase-associated
ZC3H13: zinc finger CCCH-type containing 13
FTO: fat mass and obesity-associated protein
ALKBH5: alkB homologue 5
eIF3: eukaryotic initiation factor 3
HNRNP: heterogeneous nuclear ribonucleoprotein
DUBs: deubiquitinating enzymes
Ub: ubiquitin
IHC: immunohistochemistry
HE: Hematoxylin and eosin
FBS: foetal bovine serum
MB: Methylene blue
COPS5: the fifth component of constitutive photomorphogenic-9 signalosome
YOD1: also known as OTU1, Ovarian tumor domain (OTU)-containing protein 1
BRCC3: BRCA1/BRCA2-containing complex subunit 3
VCPIP1: alosin-containing protein-interacting protein 1
SPF: specific pathogen free
RIP: RNA immunoprecipitation
IF: Immunofluorescence
OS: overall survival
DFS: disease free survival
TCGA: The Cancer Genome Atlas
MeRIP-seq: Methylated RNA immunoprecipitation sequencing
RNA-seq: RNA sequencing
KEGG: Kyoto Encyclopedia of Genes and Genomes
CCND2: cyclin D2
MEGF8: multiple epidermal growth factor-like domains 8
GLI3: glioma-associated oncogene homolog 3
CPT1A: carnitine palmitoyl-transferase 1A
ANGPTL4: angiopoietin-like 4
PLIN4: perilipin 4
TNBC: triple negative breast cancer
Abs: antibodies
CHX: cycloheximide
IB: immunoblotting
IP: immunoprecipitation
DUBs: deubiquitylases
qRT-PCR: quantitative Real-time polymerase chain reaction
IGV: Integrative Genomics Viewer
F: Forward
R: Reverse
