# Supplementary material for: Stabilization of IGF2BP1 by USP10 promotes breast cancer metastasis via CPT1A in an m6A-dependent manner
Source: Int J Biol Sci. 2023 Jan 1;19(2):449–64. doi: 10.7150/ijbs.76798 (PMC9830507; doi:10.7150/ijbs.76798)
Supplement: Supplementary file 1 — Supplementary figures and tables. [file ijbsv19p0449s1.pdf]

Supplementary figures

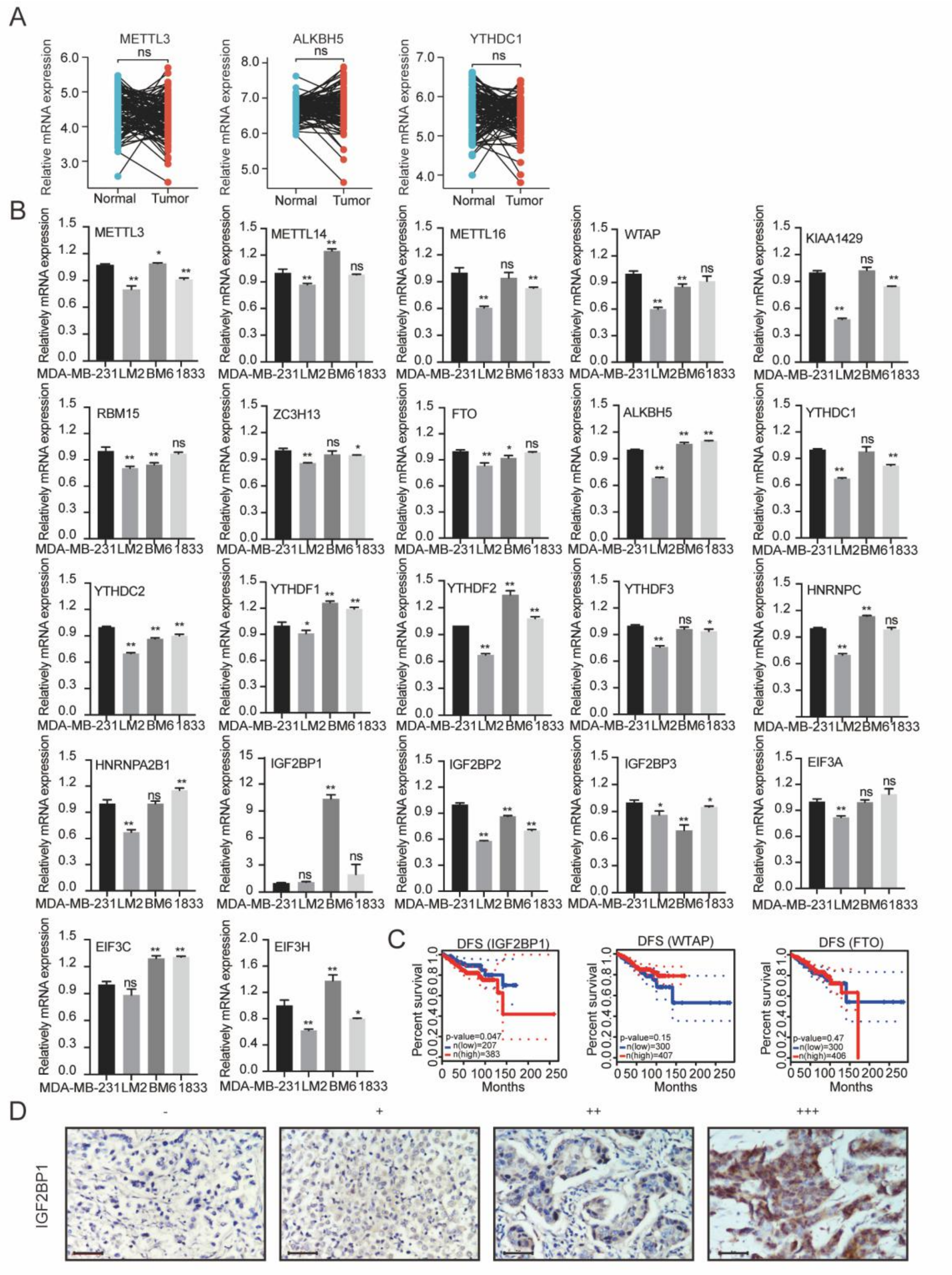

Figure S1. IGF2BP1 expression is elevated in BC.

(A) The mRNA expressions of METTL3, ALKBH5, and YTHDC1 in 112 BC tissues were compared with paired normal breast tissues from TCGA database (n=112, ns, no significance, paired t-test).

(B) The mRNA expressions of m6A-related enzymes were analyzed in MDA-MB-231, LM2, BM6, and 1833 cell lines by qRT-PCR.

(C) Kaplan-Meier survival curves of DFS based on IGF2BP1, FTO and WTAP mRNA expressions from TCGA database.

(D) Representative IHC staining images of IGF2BP1 in BC samples. Staining intensity was tagged. (scale bars=50 μm)

The data are represented as the means ± SEM. * $p<0.05$; ** $p<0.01$; *** $p<0.001$.

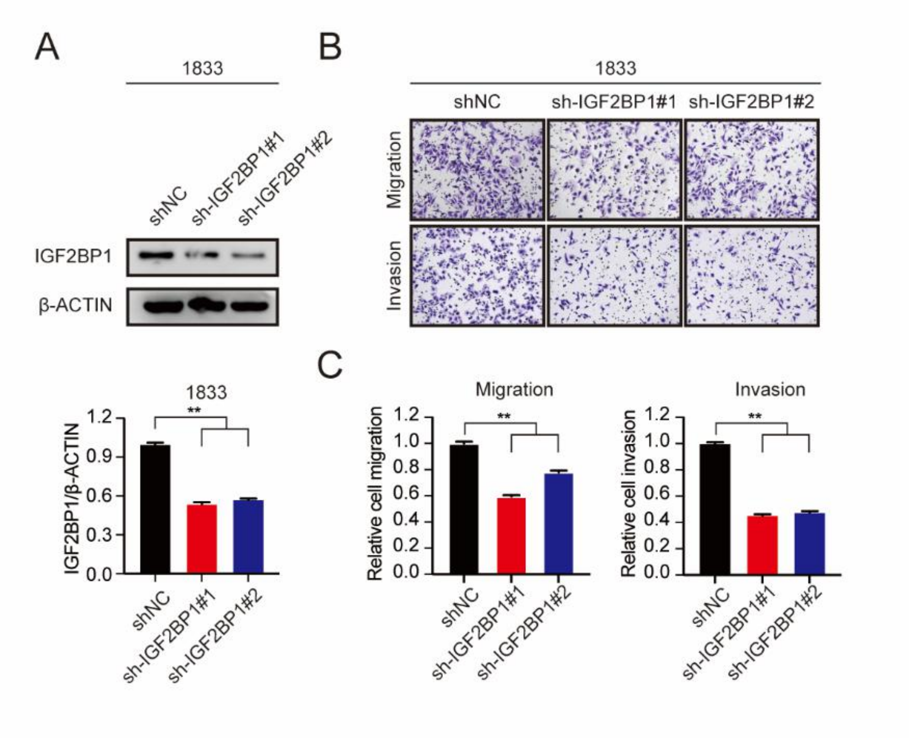

Figure S2. IGF2BP1 knockdown led to decreased cell migration and invasion abilities in 1833 cells.

(A) The efficiency of IGF2BP1 knockdown in stable IGF2BP1 downregulation 1833 cells was evaluated by western blotting (upper panel). Grayscale values were assessed by ImageJ (bottom panel).

(B-C) Migration and invasion assays of 1833 cells deficient in IGF2BP1 (B). Corresponding quantifications of the results are shown (C) respectively.

The data are represented as the means ± SEM. * $p<0.05$; ** $p<0.01$; *** $p<0.001$.

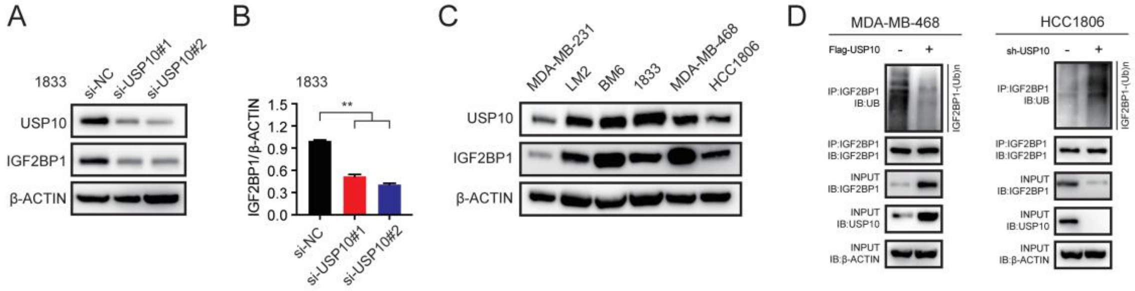

Figure S3. USP10 bound to and stabilized IGF2BP1.

(A-B) IGF2BP1 protein levels were detected by downregulating USP10 in 1833 cells (A). Densitometry quantification was determined by ImageJ (B).

(C) USP10 and IGF2BP1 protein levels in MDA-MB-231 cells, LM2 cells, BM6 cells, 1833 cells, MDA-MB-468 cells and HCC1806 cells were detected by western blot assay.

(D) USP10 was overexpressed in MDA-MB-468 cells or downregulated in HCC1806 cells followed by treatment with 10 μM MG132 for 6 h. IGF2BP1 protein was pulled down and then subjected to IB with the indicated Antibodies.

The data are represented as the means ± SEM. * $p<0.05$; ** $p<0.01$; *** $p<0.001$.

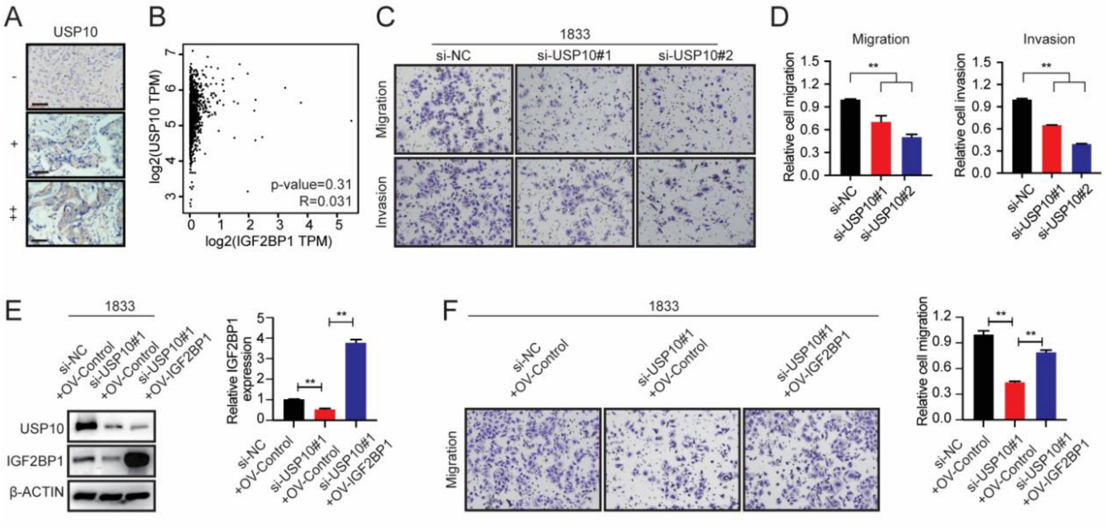

Figure S4. The USP10/IGF2BP1 axis played a pivotal role in the metastasis of BC.

(A) Representative images of USP10 IHC staining, with staining intensity tagged. (scale bars=50 μm)

(B) The correlation between the mRNA expressions of IGF2BP1 and USP10 was not significant in TCGA data.

(C-D) USP10 downregulation by specific targeting siRNAs suppressed the migration and invasion abilities of 1833 cells (C). The corresponding quantifications were also shown (D).

(E-F) IGF2BP1 overexpression rescued the inhibited ability of migration caused by USP10 knockdown in 1833 cells. Western blotting images (E) and corresponding migration assays with quantifications attached were shown (F).

The data are represented as the means ± SEM. * $p<0.05$; ** $p<0.01$; *** $p<0.001$.

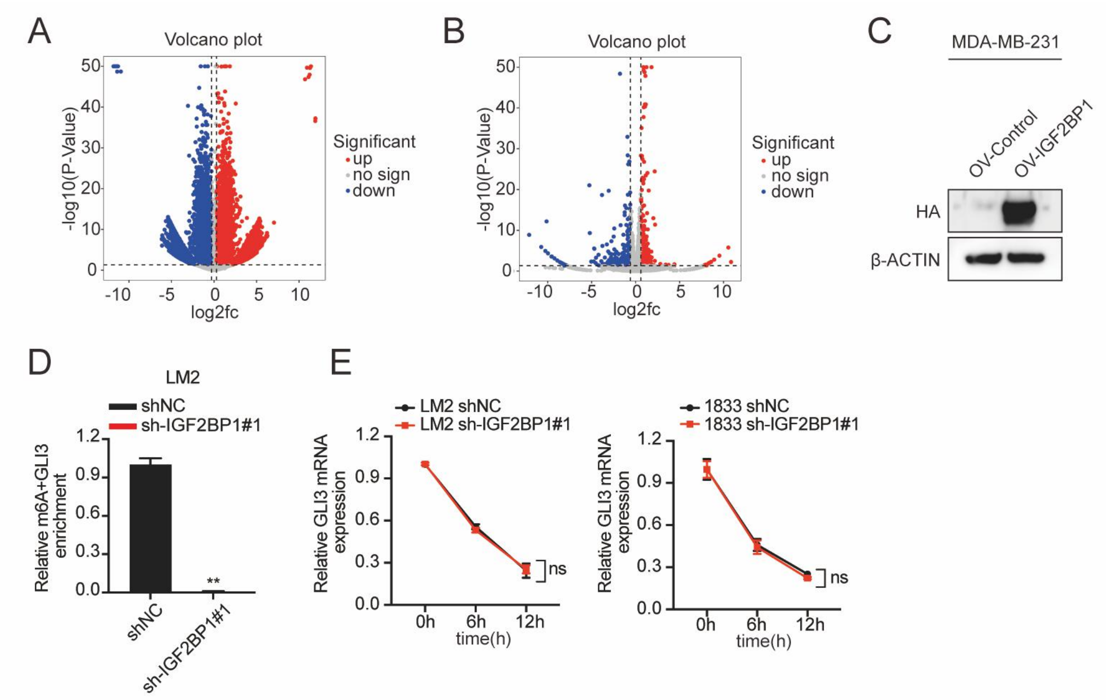

Figure S5. m6A reader IGF2BP1 stabilized CPT1A mRNA.

(A-B) The MeRIP-seq (A) and RNA-seq (B) results were shown in the volcano plots. Genes altering more than 1.5-fold change were colored.

(C) The protein level of HA-tag in MDA-MB-231 cells transfected with HA-tagged IGF2BP1 plasmids was compared with control cells.

(D) MeRIP-qPCR analysis was employed to detect the m6A modifications on GLI3 mRNA upon knockdown of IGF2BP1.

(E) The mRNA expression of GLI3 was detected in IGF2BP1 knockdown LM2/1833 cells compared with their corresponding control LM2/1833 cells treated with actinomycin D (2 μg/mL) at the indicated time points by qRT-PCR.

The data are represented as the means ± SEM. * $p<0.05$; ** $p<0.01$; *** $p<0.001$.

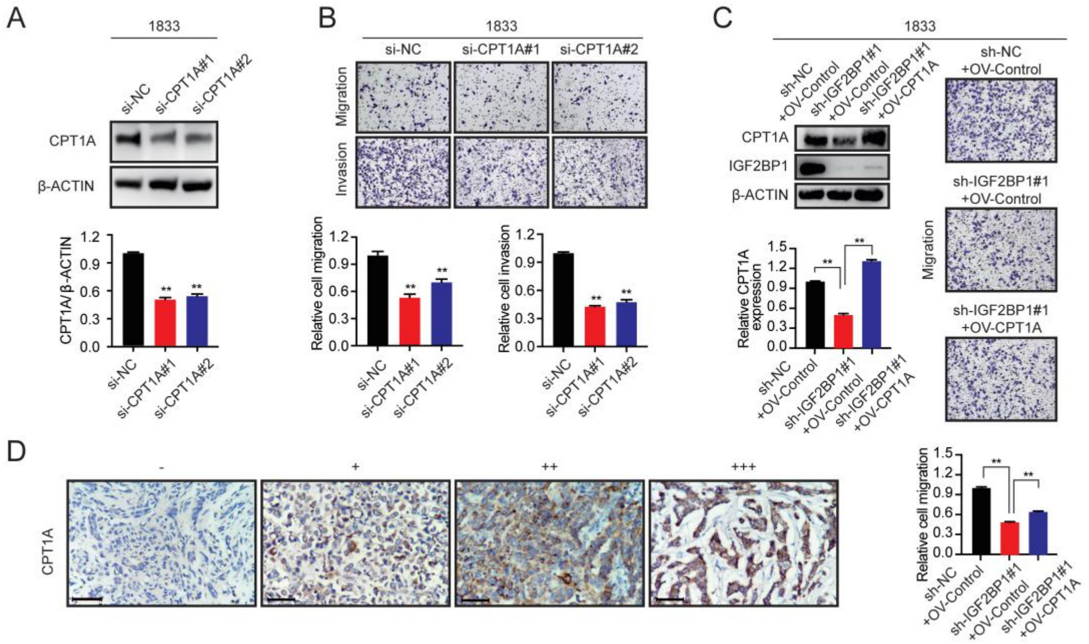

Figure S6. IGF2BP1 promoted BC metastasis via upregulating CPT1A

(A) The efficiency of CPT1A knockdown was evaluated compared with corresponding control cells by western blotting (upper panel). The quantitation results were also shown (bottom panel).

(B) Migration and invasion assays of CPT1A downregulation 1833 cells compared with corresponding control cells (upper panel). Corresponding quantitation results were also shown (bottom panel).

(C) Overexpressing CPT1A rescued the decreased migration ability caused by IGF2BP1 knockdown in 1833 cells. Western blotting images were shown. Representative images of migration assay and relative quantifications were also exhibited.

(D) Representative images of CPT1A IHC staining, with staining intensity tagged. (scale bars=50 μm)

The data are represented as the means ± SEM. * $p<0.05$; ** $p<0.01$; *** $p<0.001$.

Supplementary tables

Supplementary table 1: siRNA sequences and target sequences of lentivirals

Name	Sequence (5’-3’)
CPT1A-si-1	CCCCUCGUUAGGAAUAAUU
CPT1A-si-2	AGUCGUUCACGUUUGUUGU
CPT1A-si-3	GCCUUUACGUGGUGUCUAA
USP10-si-1	GUAUAUUUUGGAGAUUUU
USP10-si-2	GAAUAUCAGAGAAUUGAGU
USP10-si-3	CUGGAUAUUACAGCUAUUU
siRNA NC	UUCUCCGAACGUGUCACGU
shNC	TTCTCCGAACGTGTCACGT
IGF2BP1-shRNA1	CTCCAAAGTTCGTATGGTTAT
IGF2BP1-shRNA2	GCTCCCTATAGCTCCTTTATG
IGF2BP1-shRNA3	GGTGAACGAGTTGCAGAATTT
USP10-shRNA	TTCATCAGGTGGTACATCGG

Supplementary table 2: primer sequences

Name	Sequence (5’-3’)
IGF2BP1-F	GCGGCCAGTTCTTGGTCAA
IGF2BP1-R	TTGGGCACCGAATGTTCAATC
METTL3-F	ATCCCCAAGGCTTCAACCAG

METTL3-R	AGGGTGATCCAGTTGGGTTG
METTL14-F	GACGGGGACTTCATTCATGC
METTL14-R	CCAGCCTGGTCGAATTGTAC
METTL16-F	CTCTGACGTGTACTCTCCTAAGG
METTL16-R	TACCAGCCATTCAAGGTTGCT
WTAP-F	GCTTCTGCCTGGAGAGGATT
WTAP-R	GTGTACTTGCCCTCCAAAGC
KIAA1429-F	AAGTGCCCCTGTTTTCGATAG
KIAA1429-R	ACCAGACCATCAGTATTCACCT
RBM15-F	GTGAGGACTCGACTTCCCG
RBM15-R	GCCGCTATCGGTCTTTCCG
ZC3H13-F	TCTGATAGCACATCCCGAAGA
ZC3H13-R	CAGCCAGTTACGGCACTGT
FTO-F	AGACACCTGGTTTGGCGATA
FTO-R	CCAAGGTTCCTGTTGAGCAC
ALKBH5-F	ACCCCATCCACATCTTCGAG
ALKBH5-R	CTTGATGTCCTGAGGCCGTA
YTHDC1-F	AACTGGTTTCTAAGCCACTGAGC
YTHDC1-R	GGAGGCACTACTTGATAGACGA
YTHDC2-F	AGGACATTCGCATTGATGAGG
YTHDC2-R	CTCTGGTCCCCGTATCGGA

YTHDF1-F	ATACCTCACCACCTACGGACA
YTHDF1-R	GTGCTGATAGATGTTGTTCCCC
YTHDF2-F	AGCCCCACTTCCTACCAGATG
YTHDF2-R	TGAGAACTGTTATTTCCCCATGC
YTHDF3-F	TCAGAGTAACAGCTATCCACCA
YTHDF3-R	GGTTGTCAGATATGGCATAGGCT
HNRNPC-F	GGAGATGTACGGGTCAGTAACA
HNRNPC-R	CCCGAGCAATAGGAGGAGGA
HNRNPA2B1-F	ATTGATGGGAGAGTAGTTGAGCC
HNRNPA2B1-R	AATTCCGCCAACAAACAGCTT
IGF2BP2-F	AGTGGAATTGCATGGGAAAATCA
IGF2BP2-R	CAACGGCGGTTTCTGTGTC
IGF2BP3-F	ACTGCACGGGAAACCCATAG
IGF2BP3-R	CCAGCACCTCCCACTGTAAAT
EIF3A-F	GCCGGAAAATGCCCTCAAAC
EIF3A-R	TGGTTCGTGTATCTTTTGCCAT

Supplementary table 3: Antibodies for western blot, IHC, IF, and IP

Source	Item number	Name
abcam	ab208577	Anti-N6-methyladenosine (m6A) antibody [17-3-4-1]

CST	8501T	USP10 (D7A5) Rabbit mAb
CST	3724S	HA-tag (C29F4) Rabbit mAb
CST	8482S	IMP1 (D33A2) Rabbit mAb
PTG	66009-1-Ig	β -Actin Antibody
PTG	51064-2-AP	HA Tag Polyclonal Antibody
PTG	20543-1-AP	Flag-tag Polyclonal Antibody
PTG	10001-0-AP	His-tag Polyclonal Antibody
PTG	67917-1-Ig	USP10 Monoclonal Antibody
PTG	22803-1-AP	IGF2BP1 Polyclonal Antibody
PTG	15184-1-AP	CPT1A Polyclonal Antibody
yeasen	30503ES60	Flag-tag, Mouse mAb
yeasen	30701ES20	HA-tag, Mouse mAb
PTG	15073-1-AP	METTL3 Polyclonal antibody
PTG	26158-1-AP	METTL14 Polyclonal antibody
PTG	60188-1-Ig	WTAP Monoclonal antibody
PTG	27226-1-AP	FTO Polyclonal antibody
PTG	16837-1-AP	ALKBH5 Polyclonal antibody
PTG	29441-1-AP	YTHDC1 Polyclonal antibody
PTG	27779-1-AP	YTHDC2 Polyclonal antibody
PTG	17479-1-AP	YTHDF1 Polyclonal antibody
PTG	25537-1-AP	YTHDF3-specific Polyclonal antibody

PTG	11601-1-AP	IGF2BP2 Polyclonal antibody
PTG	14642-1-AP	IGF2BP3 Polyclonal antibody
